# Exploring refugees’ health care access in times of COVID-19: a quantitative study in the Lisbon region, Portugal

**DOI:** 10.3389/fpubh.2024.1337299

**Published:** 2024-01-29

**Authors:** Vanessa Portela, Sousan Hamwi, Maria R. Oliveira Martins

**Affiliations:** ^1^Institute of Hygiene and Tropical Medicine, NOVA University of Lisbon, Lisbon, Portugal; ^2^NOVA National School of Public Health, Public Health Research Centre, NOVA University of Lisbon, Lisbon, Portugal; ^3^Global Health and Tropical Medicine, Institute of Hygiene and Tropical Medicine, NOVA University of Lisbon, Lisbon, Portugal

**Keywords:** refugees, migrants, health care access, COVID-19, Portugal

## Abstract

**Background:**

To address the health needs of refugees, health services must be culturally competent and facilitate this population’s access to health care, especially in a context prone to the amplification of social inequities, such as the COVID-19 pandemic. However, few quantitative studies exist in the European Union, and to the extent of our knowledge, there are no published quantitative studies exploring refugees’ access to health care during the pandemic in Portugal. The objective of this study is to describe the demographic and socioeconomic characteristics of refugees living in Lisbon and to explore their healthcare access patterns during the COVID-19 pandemic.

**Methods:**

We conducted a cross-sectional, descriptive, and quantitative study from May to November 2022. Using Levesque’ s theory on health care access, we designed and applied a 38-item questionnaire through face-to-face interviews with refugees living in Lisbon for at least 12 months, and used descriptive statistics to characterize sociodemographic and healthcare access profiles during the pandemic.

**Results:**

The mean age of the 36 recruited refugees was 35 years (SD = 10.24), the majority were male (56%), married (72%), had at least a secondary education (69%), were unemployed (77.8%), and had a median length of stay in Portugal of 17 months (IQR = 45). All were registered in a primary care center, and 94% used healthcare services during the pandemic. The majority never tested positive for the coronavirus (58%) and one out of the positive was admitted to hospital due to severe COVID-19. A total of 97% received COVID-19 vaccination, of which 69% had an incomplete schedule. A quarter of the participants did not have access to information about COVID-19 in a language they understood, and although 97% needed health care during the pandemic, more than half (63%) did not seek it because of structural and cultural barriers. Half of the respondents had difficulty getting medical advice by phone or email, and 39.4% could not afford a medical examination or treatment. Only 18.2% sought counseling services. A total of 58.8% of the participants felt like healthcare professionals did not always show respect towards their culture, and 64.7% reported that healthcare professionals did not always discuss treatment options with them.

**Conclusion:**

This study’s findings highlight the need to endow inclusive communication, cultural competency, and patient involvement in health care, alongside improving the socioeconomic condition of refugees. Identified population characteristics and barriers to health care access by refugees in this study may inform future research on the health care needs of refugees in Portugal and ultimately assist in the devising of strategies to reduce inequalities in health care access.

## 1 Introduction

With the increasing global migratory flows, health care access by migrant populations has been a subject of international and national research. The concept of access to health care in the literature encompasses diverse definitions. Some definitions view access as the attributes of health services, users, or both, while others focus on the relationship between the supply and demand for health care (1). Levesque’s Conceptual Framework for Heath Care Access analyzes access as a product of the relationship between five dimensions of accessibility of services (Approachability, Acceptability, Availability and Accommodation, Affordability, and Appropriateness) and five abilities of persons (Ability to perceive, Ability to seek, Ability to reach, Ability to pay, and Ability to engage) (2). This multidimensional and integrative approach of the theory may provide means to characterize health care access more comprehensively and accurately. Long-standing barriers to health care access by refugees and migrants are robustly documented in the literature. Nested within cultural, social, and financial factors, lie obstacles such as language differences, discrimination, unawareness about individual rights to health or available healthcare services, economic insufficiency and out-of-pocket expenditures (3), bureaucracy and lack of refugee documentation status, and long distances to healthcare facilities (3–6).

The additional burden of the COVID-19 pandemic on refugees’ social determinants of health through the loss of jobs, increased poverty, discrimination, and social isolation, along with COVID-19 prevention and control measures, led to a disproportionate impact of the pandemic on this vulnerable group (7). Constraints in services such as childcare, language classes, and provision of resettlement services (8) accounted for the weakening of social support networks. Pre-existing healthcare barriers further compromised refugees’ access to health care, namely in accessing COVID-19 information (9) or testing (10). Social distancing and lockdowns enhanced the reliance on technology for the delivery of health care services. Studies with refugees show that while the use of technology provided advantages in some aspects of access (i.e., users’ convenience in travelling-associated costs and time, especially for people living in remote areas) (11), it also presented several challenges. In this context, a different set of health care access barriers emerged, such as technology costs and complexity, technical and operational issues (i.e., connectivity problems), interference with quality of care (communication and development of a trustful relationship with providers) (11), lack in technology literacy, issues with communication and cultural mediation services, and privacy concerns (8).

In Portugal, there are published studies on health care access and utilization by the overall immigrant population, which show the presence of systemic barriers both before and during the pandemic. In a 2018 study involving 1,375 immigrants and 320 professionals from primary care centers in Lisbon, Dias et al. explored the perceptions of both groups on the access and utilization of healthcare services. Economic, cultural, linguistic, and discriminatory obstacles were identified (12). These constraints led to an underutilization of healthcare services by the immigrant population (13–16). A 2021 survey on health care access by immigrants in Portugal underscored the unmet needs for medical care due to financial constraints, long waiting lists, lack of time due to occupational or family responsibilities, dissatisfaction, and lack of trust in public healthcare services (17). O Martins et al. through a cross-sectional study in Lisbon’s Metropolitan Area, highlighted the disproportionate socioeconomic impact of the COVID-19 pandemic on immigrants compared with natives. Findings revealed that COVID-19’s effects amplified immigrants’ previous hardships leading to greater job loss, lay-offs, and income losses, with a consequential impact on livelihoods. Moreover, in the early stages of the pandemic, immigrants had increased difficulties in accessing healthcare services in comparison with natives. Immigrants were more likely to face hindrances in obtaining medical appointments, in complying with children’s vaccinations, and in the acquisition of pharmaceuticals (18, 19).

Although there has been a substantial increase in the number of people in need of international protection in Portugal since 2015 (20), little is known about refugees’ reality, namely in what concerns their health care. Despite the aforementioned research on health care access by the overall immigrant population in Portugal, to the extent of our knowledge, there are no published quantitative studies addressing this issue in refugees, particularly during the COVID-19 pandemic. As such, this study may provide valuable initial information about the subject.

Although in Portugal asylum seekers and refugees’ rights to health care are enshrined in the national Asylum Act (21), it is essential to understand if this translates into an effective and equitable access to health care, particularly in the midst of a pandemic. Furthermore, as other migrants in vulnerable situations, assurance of access to health care by refugees is imperative, as their needs differ from the host population due to the cumulative effect of risk factors to poor health, which act throughout the migration process (22). Therefore, host countries need to define evidence-based interventions that protect this vulnerable group’ s health.

Within this context, we may ask: what have been the main difficulties in health care access by refugees in Lisbon during the COVID-19 pandemic? The objective of our study is to describe the demographic and socioeconomic characteristics of refugees living in Lisbon and to explore their health care access patterns during the COVID-19 pandemic.

The outcomes of this study will potentially provide preliminary information about barriers experienced by refugees, which may serve as a basis to larger studies, and assist in the devising of vertical health policies to improve their health care access.

## 2 Materials and methods

### 2.1 Study design and setting

We conducted a cross-sectional, descriptive, and quantitative study in Lisbon, between May and November 2022. We found this type of study the most suited to explore the unknown reality of health care access among refugees in Portugal and to address the multidimensional and multivariable nature of the chosen framework on health care access, while saving time and resources.

Although the reception and geographical distribution of asylum-seekers and international protection beneficiaries in Portugal is tendentially decentralized, Lisbon has been the district receiving the greatest number of refugees through all programmed entry mechanisms globally (2018–2021) (20). To recruit the participants, we collaborated with the community intervention organization CRESCER, within the scope of the organization’s projects “É UMA VIDA [IT’S A LIFE]” and “NO Border.” CRESCER develops assistance projects for vulnerable populations in the greater Lisbon area. Since 2016, they have also cooperated with Lisbon’s Municipal Refugee Reception Program (PMAR Lx) during its second phase, facilitating the transition of refugees and asylum seekers from the Refugee Reception Center to temporary autonomous housing granted by the municipality. Additionally, CRESCER provides assistance in areas like employment search, legal and psychosocial support, medical and psychological care, housing, mediation, and translation. This comprehensive support is delivered by a technical team of social workers, interpreters, a psychiatrist, a psychologist, and a lawyer (23).

### 2.2 Participants and sampling

The target population was adult refugees as defined by the United Nations Convention relating to the Status of Refugees (24). Participation in the study required the fulfillment of the eligibility criteria, which consisted of being a refugee, aged 18 or more, living in Lisbon, with a length of stay in Portugal of at least 12 months, and receiving assistance from the community intervention association CRESCER. From late April to November 2022, CRESCER’s professionals contacted potential participants during the social support appointments at the organization’s headquarters or during the technical teams’ weekly home visits. Individuals were excluded from the study if there was no interpretation available for their languages. For this exploratory study, we used a non-probabilistic convenience sample, as it was the most cost-effective method to meet the study’s objectives within a short period. Interviews were conducted at the home of the “É UMA VIDA” project beneficiaries or CRESCER’s headquarters in the case of the “NO Border” project recipients. In all cases, the interview place was chosen considering the participants’ convenience and in alignment with CRESCER’s engagement context for each project.

### 2.3 Measurement instrument and variables

**Figure 1:**
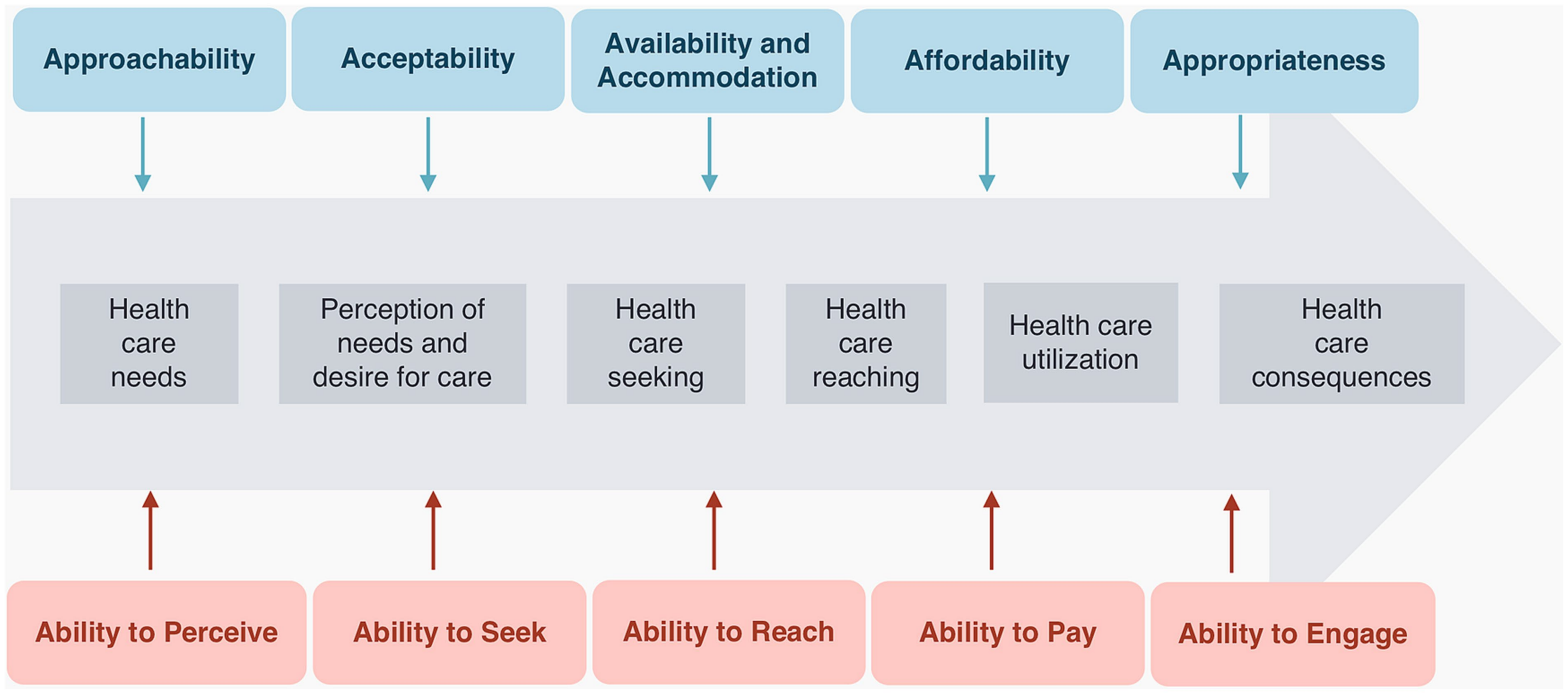
Levesque’s framework of access to health care Adapted from: patient-centred access to health care: conceptualizing access at the interface of health systems and populations, Levesque et al. (2) (p.5).

We designed a structured 38-item questionnaire, which was translated from Portuguese into English and Arabic by the research team and interpreted into the other languages of the refugees in the sample (i.e., Kurdish and French), with the collaboration of the organization’s cultural mediators. The questionnaire referred to the period between the beginning of the COVID-19 pandemic in Portugal (March 2020) and the moment of the interview and was structured in two domains for content organization and analysis facilitation purposes: (1) sociodemographic-, migration-, healthcare services-, and COVID-19-related variables and (2) dimensions of access to health care, using Levesque’s theory on health care access. Levesque’s theory identifies two main components: “accessibility of services” and “abilities of persons.” The “accessibility of services” encompasses five dimensions: Approachability, Acceptability, Availability and Accommodation, Affordability, and Appropriateness. Correspondingly, the “abilities of persons” include the abilities to Perceive, to Seek, to Reach, to Pay, and to Engage in healthcare. The dimensions of “accessibility of services” interact with the corresponding dimensions in “abilities of persons” to generate access (2), as shown in Figure 1. This multidimensional approach and the holistic view of access illustrate the complex interactions that generate access and stand for the comprehensive nature of the theory. Drawing on Levesque’s framework, we selected variables from established public health surveys, with the intent of capturing the determinants of all health care access dimensions among refugees during the COVID-19 pandemic, albeit with a focus on the abilities of persons. We classified the variables into the dimensions they best reflect, according to the categorization used in the literature and the research team’s interpretation of the framework, as follows:

#### 2.3.1 Approachability

We explored this dimension using the determinant “information.” Namely, we assessed whether health services conveyed information about COVID-19, considering audiences from diverse cultural backgrounds. By making information culturally adequate and available in different languages, recipients can identify and use healthcare services according to their health needs. For example, clear information in several languages about COVID-19 testing procedures could enhance testing adherence among refugees not proficient in Portuguese or English.

#### 2.3.2 Ability to perceive

This ability was analyzed through the determinants “health literacy” and “health beliefs,” both of which influence the individual’s perception of health issues and the realization of the need for care. We used the variables “source of information about COVID-19,” “knowledge of symptoms of COVID-19,” and “asymptomatic spread of COVID-19” to assess health literacy, the variable “prevention of COVID-19 by eating spicy food” to assess health beliefs, and the variable “need for health care during the pandemic” to explore both.

#### 2.3.3 Acceptability

We used the variable “cultural competence in health care provided” to explore this dimension. For health care to be accepted, the provision of services must be culturally adequate to engage users in seeking care. Likewise, healthcare professionals need to be equipped with skills that stimulate cultural awareness when delivering care to people from diverse backgrounds.

#### 2.3.4 Ability to seek

The variables “sought health care every time needed” and “reasons for not seeking health care during the pandemic” were used to assess the determinants of autonomy, personal and social values, and individual rights. Understanding why individuals in need of care do not seek it helps identify barriers to their autonomous decision-making regarding seeking healthcare. To explore users’ awareness of different health care modalities, we examined the variables “type of healthcare providers sought during the pandemic,” “type of healthcare services sought during the pandemic,” and “knowledge about line SNS 24″. The awareness of “line SNS 24″ (Portuguese national health system phone and online platform) was particularly important during the pandemic. This line was designated as the primary point of contact between the public and the health system, alongside providing advice and guidance on COVID-19 preventive measures, symptoms, contacts, testing, quarantine, and when necessary, referral for medical observation. Therefore, awareness of its existence was essential to access some key healthcare services.

#### 2.3.5 Availability and accommodation

With the establishment of public health measures (such as social distancing) and with the need to avoid health system saturation during the pandemic, healthcare services were required to diversify the ways of providing care, by swiftly investing in remote or virtual modalities of contact with users. Thus, the variable “get medical advice by email/phone” was chosen to assess the availability of alternative methods to in-person healthcare service provision.

#### 2.3.6 Ability To reach

This ability was analyzed through the variable “travel to healthcare facility” which explores the easiness with which people can get to the healthcare unit in case of need. It is determined by the concept of personal mobility and availability of transport (2). Another important determinant to reach health care is occupational flexibility, which was assessed by “medical appointment/perform exams during working hours.”

#### 2.3.7 Affordability

The variable “pay for healthcare services” was used to assess the direct costs of services, namely if refugees were required to pay for any healthcare services.

#### 2.3.8 Ability to pay

This ability translates to the economic capability to pay for health care without incurring expenses that endanger the supply of basic needs (2). It was analyzed through the variable “could not afford medical examination/treatment.”

#### 2.3.9 Appropriateness

The determinant adequacy of care was assessed using the variable particularly if the provided healthcare addressed the specific linguistic needs of refugees. The variable “discussion of treatment options/side effects” was used to assess the technical and interpersonal quality of care, namely if healthcare professionals provided holistic information about treatment options and involved refugees in treatment decisions.

#### 2.3.10 Ability to engage

The variables “vaccination against COVID-19” and “preventive measures SARS-CoV-2” were used to analyze the determinant adherence, namely if refugees were involved in COVID-19 precautionary actions.

### 2.4 Ethical considerations

The study’s protocol was approved by the Ethics Committee of Institute of Hygiene and Tropical Medicine, NOVA University of Lisbon (IHMT) and guided by the ethical principles of the Declaration of Helsinki (25). A written informed consent was obtained from the individuals to participate in the study, which consisted of answering a face-to-face questionnaire applied by the research team. Participation in the study was voluntary, and data was treated confidentially and anonymously. The informed consent was translated from Portuguese into English, Arabic, and French by the research team, and into Kurdish by a cultural mediator from CRESCER, to allow a comprehensive understanding of the information. Whenever the participant was illiterate, the informed consent was read by the cultural mediator in the participant’s language in the presence of a witness (usually a family member). Participants’ privacy, in the case of the “NO Border” project, was ensured by conducting the interviews in a separate room at the organization’s headquarters.

### 2.5 Statistical methods

We summarized data using descriptive statistics: we computed frequencies and proportions for qualitative (nominal and ordinal) variables, and the mean and standard deviation (SD), or the median and interquartile range, for quantitative variables, according to their distribution. A database was created and analyzed using the IBM®SPSS® Statistics version 28.

## 3 Results

### 3.1 Participants

A total of 37 refugees were identified by CRESCER as meeting the eligibility criteria for the study and were invited to participate. One participant declined participation due to a lack of time to answer the questionnaire, resulting in a final sample size of 36.

### 3.2 Socioeconomic and demographic characteristics

As shown in Table 1, the mean age of the participants was 35 years (SD = 10.24 years), with over half being male (*n* = 20, 55.6%), 26 (72.2%) were married, and the majority had an Islamic religious background (n = 25, 69.4%). The participants were from seven countries across the Middle East, Asia, and Africa (mainly Afghanistan, Iraq, and Syria) and had all been integrated into the government’s Refugee Reception Program. The median length of their stay in Portugal was 17 months (IQR = 45) by the time the questionnaire was applied. Most participants had at least a secondary school education (*n* = 25, 69.4%), and were not verbally proficient in Portuguese (*n* = 23, 63.9%) or in English (*n* = 27, 75%). In six interviews where the house representatives were fluent in English, they acted as an interpreter for the other family member(s) included in the study. Concerning employment, the majority of participants were unemployed (*n* = 28, 77.8%), including nine (25%) housewives. With a median of four persons living in the same household (IQR = 4), all the participants expressed some degree of difficulty making ends meet, of which 19 (52.8%) indicated great difficulty. All 36 participants were registered in a primary healthcare center and, during the pandemic, 34 (94.4%) used healthcare services. Regarding infection with SARS-CoV-2, only two participants were never tested during the study period. Of those tested, the majority never tested positive (n = 21, 58.3%), while a total of 13 (36.1%) tested positive at least once. Among the latter, only one required hospitalization due to severe COVID-19 symptoms.

**Table 1:** 1st domain: sociodemographic-, migration-, healthcare services-, and COVID-19-related variables.

VARIABLE	FREQUENCY *(n)*	%
*Employment status*		
Unemployed	19	52.8
Housekeeper	9	25.0
Employed	8	22.2
*Number of people in household (Median, IQR)*		
	4 (IQR = 4)	
*Integration Refugee Reception Program (yes)*		
	36	100
*Native language*		
Arabic	10	27.8
Kurdish	10	27.8
Dari	8	22.2
Pashto	5	13.9
Tigrinya	2	5.6
French	1	2.8
*Portuguese verbal proficiency*		
not at all	2	5.6
not well	21	58.3
well	12	33.3
very well	1	2.8
*Primary Care Center registration (yes)*		
	36	100
*Length of stay in Portugal, months (Median, IQR)*		
	17 (IQR = 45)	
*Healthcare services utilization (yes)*		
	34	94.4
*Test positive for coronavirus*		
no	21	58.3
yes	13	36.1
never been tested	2	5.6
*Admission to hospital due to COVID19*		
no	12	33.3
yes	1	2.8
never been tested/never tested positive	23	63.9
*Vaccination against COVID-19*		
no	1	2.8
yes, but not all doses required	25	69.4
yes, all doses required	10	27.8
*COVID-19 Preventive measures*		
wore face mask	36	100
used sanitizers	35	97.2
washed hands for 20 s	33	91.7
kept social distance	30	83.3
did not touch my face	13	36.1
changed my diet	7	19.4
took over-the-counter medicines	4	11.1
other preventive measures	1	2.8

### 3.3 Health care access profiles during the COVID-19 pandemic

#### 3.3.1 Approachability and ability to perceive

In this study, 25% of the participants reported not accessing information about COVID-19 in a language they understood. Almost a third of the participants relied on social media and family/friends to obtain information (*n* = 11, 30.5%). Almost all participants (*n* = 35, 97.2%) needed some kind of health care during the pandemic. Most participants (over 86%) were able to identify the most common symptoms of COVID-19 (i.e., fever/chills, cough, fatigue, loss of taste and/or smell), and over 69% recognized three of the less common symptoms (i.e., muscle/body aches, sore throat, congestion/runny nose) (26). However, the identification of gastrointestinal symptoms, specifically nausea or vomiting and diarrhea, varied among participants, with only 44.4 and 38.9% recognizing them correctly (respectively). When presented with false symptoms, 22 (61.1%) correctly identified constipation as unrelated to COVID-19, while 26 (72.2%) did the same for bleeding. Most participants (*n* = 21, 58.4%) either did not know or incorrectly believed that an asymptomatic person could not spread the virus. Additionally, one-sixth (*n* = 6, 16.7%) held the misconception that COVID-19 can be prevented by eating spicy food.

#### 3.3.2 Acceptability and ability to seek

As shown in Table 2, when receiving health care, 58.8% of the participants felt like healthcare professionals did not always show respect towards their culture, including 17.6% who rarely or never felt respected. More than half of the participants (*n* = 22, 62.9%) did not seek health care every time they needed it. Of the mentioned reasons for not seeking health care, most were related to the difficulty of getting a medical appointment, whether due to a long waiting list (*n* = 16, 72.7%) or because the appointment got canceled/postponed (*n* = 6, 27.3%). Language difficulties were pointed out by 13 (59.1%) respondents, and 12 (54.5%) considered their health problem not to be serious enough to justify seeking health care. A total of 10 (45.4%) respondents did not know what to do or where to go for health care. The main healthcare providers sought by the participants during the pandemic were family doctors (*n* = 23, 69.7%), pharmacists (*n* = 19, 57.6%), hospital specialist doctors (*n* = 17, 51.5%) and emergency rooms (*n* = 17, 51.5%). Only six (18.2%) sought counseling services. Most participants relied on the public sector, namely primary care centers (*n* = 27, 81.8%) and public hospitals (*n* = 26, 78.8%), to get health care. Half of the participants (*n* = 18, 50%) were unaware there was a national health line (linha SNS24).

**Table 2:** Acceptability and ability to seek results.

Dimension/Question	Responses *N* = 36			
	Always *n* (%)	Sometimes *n* (%)	Rarely *n* (%)	Never *n* (%)
*Acceptability*				
*When receiving health care during the pandemic, did you feel that health care professionals were understanding and respectful of your culture?*	14 (41.2)	14 (41.2)	5 (14.7)	1 (2.9)
	*n* (%)			
*Ability to Seek*				
*During the pandemic, did you seek health care every time you needed it?*				
no	22 (62.9)			
yes	13 (37.1)			
*If you did not always seek health care whenever you needed it, please indicate why*				
Could not make an appointment because of long waiting list	16 (72.7)			
Language difficulties	13 (59.1)			
My health problem was not serious	12 (54.5)			
Did not know what to do	7 (31.8)			
Appointment got canceled/postponed	6 (27.3)			
Could not afford health care	6 (27.3)			
Fear of getting COVID-19	6 (27.3)			
Preferred to seek traditional/alternative medicine from my country of origin	6 (27.3)			
Do not trust healthcare professionals	4 (18.2)			
Did not know where to go	3 (13.6)			
Fear of discrimination	3 (13.6)			
Did not know if I was entitled to health care	3 (13.6)			
Did not have means of transportation	2 (9.1)			
Fear of denunciation due to my legal situation	0 (0.0)			
Other	2 (9.1)			
*During the pandemic, which healthcare providers did you seek? (select all that apply)*				
Family medicine doctor	23 (69.7)			
Pharmacist	19 (57.6)			
Hospital specialist doctor	17 (51.5)			
Emergency room	17 (51.5)			
Dentist	14 (42.4)			
Nurse	7 (21.2)			
Psychological & counseling services	6 (18.2)			
Traditional healer	1 (3.0)			
*During the pandemic, which health services did you seek?*				
Primary care center	27 (81.8)			
Public hospital	26 (78.8)			
Private clinic/hospital	7 (21.2)			
Non-governmental organization	5 (15.2)			
Other	1 (3.0)			
*Do you know what is the health line SNS24 (linha SNS24)?*				
No	18 (50)			
Yes	18 (50)			

#### 3.3.3 Availability and accommodation and ability to reach

Of the 26 respondents who contacted the healthcare center by phone or email, half (n = 13, 50%) found it was very difficult to get medical advice through those channels of communication. When considering the physical mobility to the health center, most participants reported that it was very easy (n = 17, 51.5%) or somewhat easy (n = 9, 27.3%) to get to the primary care center or hospital. Out of the six employed refugees who needed care during work hours, four had the occupational flexibility to go to a medical appointment or perform an exam.

#### 3.3.4 Affordability and ability to Pay

When it comes to the direct costs of health care, over half of the participants (*n* = 19, 54.5%) reported having paid for health care (including medication). A total of 13 (39.4%) respondents experienced times when they could not afford a medical examination or treatment.

#### 3.3.5 Appropriateness and ability to engage

Table 3 reports the dimensions of appropriateness and the ability to engage. Over a third of the participants (*n* = 11, 35.5%) were not offered an interpreting service when receiving health care, and for the ones who were, interpretation was provided by the organization CRESCER or a family member proficient in English. A total of 22 (64.7%) participants reported that when receiving health care during the pandemic, healthcare professionals did not discuss with them treatment options or treatment side effects. Most participants (*n* = 35, 97.2%) had received vaccination against COVID-19, of which 25 (69.4%) had an incomplete vaccination schedule. In what concerns COVID-19 preventive measures, all the participants wore face masks, and the large majority (*n* = 30, 83.3%) used sanitizers, washed their hands for 20 s, and kept social distance. Around 30% of participants changed their diet or took over-the-counter medicines to protect themselves from SARS-CoV-2 infection.

**Table 3:** Appropriateness and ability to engage results.

Dimension/Question	Responses *N* = 36			
	*n (%)*			
*Appropriateness*				
*When receiving health care were you ever offered an interpreting service?*				
No	11 (35.5)			
Yes	20 (64.5)			
	*Always* *n (%)*	*Sometimes* *n (%)*	*Rarely* *n (%)*	*Never* *n (%)*
*When receiving health care during the pandemic, have healthcare professionals discussed with you your different treatment options, including possible side effects?*	12 (35.3)	11 (32.4)	3 (8.8)	8 (23.5)
	**n (%)**			
*Ability to engage*				
*Were you vaccinated against COVID-19?*				
No	1 (2.8)			
Yes, but not all doses required	25 (69.4)			
Yes, all doses required	10 (27.8)			
*COVID-19 Preventive measures*				
Wore face mask	36 (100)			
Used sanitizers	35 (97.2)			
Washed hands for 20 s	33 (91.7)			
Kept social distance	30 (83.3)			
Did not touch my face	13 (36.1)			
Changed my diet	7 (19.4)			
Took over-the-counter medicines	4 (11.1)			
Other preventive measures	1 (2.8)			

## 4 Discussion

### 4.1 Sociodemographic characteristics

This article aimed to describe the sociodemographic, migration, and COVID-19 characteristics of refugees living in Lisbon, and to describe the dimensions of their health care access during the COVID-19 pandemic in consistent with Levesque’s Patient-Centered Framework. A total of 36 refugees participated in the study, with a mean age of 35 years, and over half were male (*n* = 20). The participants were from seven countries across the Middle East, Asia, and Africa, had all been integrated into the government’s Refugee Reception Program, and had a median length of stay in Portugal of 17 months. Of the 36 participants, 26 were married, with a median of four persons living in the same dwelling, and the majority had an Islamic religious background (*n* = 25). Most had at least a secondary school education (*n* = 25) and were not verbally proficient in Portuguese (*n* = 23) or English (*n* = 27). The large majority were unemployed (*n* = 28) and all expressed some degree of difficulty making ends meet. All were registered in a primary healthcare center and, during the pandemic, 34 used healthcare services. Most of them never tested positive for SARSCoV-2 (*n* = 21), and one was admitted to hospital due to severe COVID-19.

Sociodemographic data on refugees and asylum seekers resettled in Portugal is dispersed and often incomplete. According to available national data from the last 5 years (which corresponds to the longest length of stay of this study’s participants), the sociodemographic distribution of refugees was overall similar to that in this study’s sample. For example, most refugees - including children - were male (around 68% in 2018, around 60% in 2020 and 52% in 2021 compared to 55.6% in the sample) (20, 27). Additionally, the most representative age group among adult refugees (over 18) was 19–39 years (89% in 2018, 78% in 2019, 76% in 2020, and 66% in 2021) (20), similar to the age distribution of the study (in which n = 27, 75% aged 21–38 years). The most frequent countries of origin (Afghanistan, Iraq, Syria, and Eritrea) are also among the main five countries of origin of refugees and asylum-seekers in Portugal in the last 5 years (27). National data concerning the educational attainment of refugees is limited. In 2021, the educational level of up to 40% of refugees arriving in Portugal was unknown, including those from Afghanistan—the most prevalent group at the time. For those whose educational level was documented, most had only completed primary school. This contrasts with the findings of our study, where the majority had at least a secondary school education (20). Regarding employment, in 2020 and 2021, around 40% of the refugees were still unemployed at the end of the integration program, whilst in our study, unemployment almost reached an astounding 78%. Similarly to the findings of this research, there were high percentages of registration of newly arrived refugees in the National Health System (SNS) in 2021, namely over 80% in all official entry mechanisms (except for the Afghans, which was 69.4%) (20). Although our sample is not intended to be representative, a comparison with the limited data available at the national level shows that the age, gender, and country of origin of the refugees in our study do not differ considerably from the national picture.

### 4.2 Approachability and ability to perceive

When considering healthcare services’ approachability, especially in public health emergencies such as the COVID-19 pandemic, services must devise strategies of communication in which information is available, clear, and adequate to the audience it serves, so that preventive and control measures can be promptly and efficiently followed. Inclusive communication in health care should take into consideration not only the diverse cultural backgrounds of the recipients (namely by making information culturally adequate and available in different languages) but also ensure that it reaches the intended public promptly, so that services can be identified by users. Although several entities such as the Directorate-General of Health, non-governmental organizations, the International Organization for Migration, and particularly the High Commission for Migration publicized multilingual information about COVID-19, 25% of the participants in this study stated not to have had access to information about COVID-19 in an understandable language. Studies in countries such as the United Kingdom and Brazil similarly showed that there was insufficient communication effectiveness with asylum seekers, due to a lack of culturally and linguistically adequate information about the pandemic (28).

The Ability to perceive the need for care is highly influenced by health literacy, knowledge, and beliefs about health (2). The main sources of information on COVID-19 chosen by the participants were informal, such as social media and friends/family, which accounted for almost a third of the responses. This finding is in line with other studies, which outline the role of social media as a source of information about COVID-19 (29–31). Factors such as age, language proficiency, education, economic resources, and length of stay in the country may all have played a part in the choice of the information source. Healthcare services that are not approachable, due to their failure to convey culturally and linguistically appropriate information, can negatively affect people’s ability to access and perceive reliable health information. People may then turn to sources of information that are readily available and free of language barriers, like social media, as outlined in a systematic review on the use of social media during the pandemic by ethnic minorities and migrants (including refugees) (31). Social media channels may, in turn, become a vehicle for health misinformation, particularly during public health emergencies like the COVID-19 pandemic, with negative impacts on people’s health behavior, such as increasing vaccine hesitancy and the use of unproven treatments (31–33).

In this study, most participants were knowledgeable about the most common symptoms of COVID-19, which can be partially explained by the high level of education of the participants and the study’s timing (*circa* 2 years of pandemic). Nevertheless, a lack of awareness about asymptomatic transmission of the virus was also common, and up to one-sixth of respondents held the misconception that COVID-19 can be prevented by eating spicy food. The literature on the levels of knowledge about transmission and symptoms of COVID-19 among forcibly displaced people is heterogeneous but generally shows that lower levels of knowledge and health literacy are more likely in refugees with low educational attainment (30, 34–36). Other factors that probably influence knowledge levels include the different study settings (camps versus urban resettlements), timing of the studies/time elapsed since the beginning of the pandemic (and thus production of knowledge about the novel virus), and language proficiency.

### 4.3 Acceptability and ability to seek

When receiving health care, most participants of this study felt like healthcare professionals did not always show understanding and respect towards their culture, including almost a fifth who rarely or never felt culturally respected. This finding elicits a lack of cultural competence, which is defined as the ability of systems to provide care to patients with diverse values, beliefs, and behaviors, including tailoring delivery to meet patients’ social, cultural, and linguistic needs (37). Lack of cultural competence in the provision of healthcare services compromises its acceptability by users (38). Cultural and religious differences between participants and their healthcare providers may have played a role in this dimension, as most refugees were of Islamic background, in contrast with the predominant Christian-embedded culture of Portugal. There is a paucity of data in the literature on refugees’ input on cultural competence in the healthcare setting (39). Findings of the qualitative arm of a European study on the healthcare of migrants and refugees highlighted a perceived cultural competence inadequacy among healthcare providers in all ten participating countries (Portugal not included) (40). Similarly, studies with healthcare professionals in Portugal acknowledged the cultural challenges in providing care to migrants, including the lack of cultural competence training (41) and the need to incorporate cultural mediators in healthcare services (42, 43). Healthcare professionals’ lack of awareness and preparedness regarding certain cultural aspects of refugees and other migrants may lead to feelings of rejection and imperil health care access through the avoidance of healthcare providers (3, 44), thus endangering acceptability. Additionally, disrespect towards the culture of migrants and refugees, in the form of discrimination or xenophobia, is also a well-known barrier to health care (3, 38, 40, 45), further compromising access. Results from a training program on cultural and individual diversity for primary healthcare providers in Portugal during the COVID-19 pandemic showed an improvement in cultural diversity awareness, knowledge, and skills, and contributed to reducing feelings of discrimination among healthcare professionals (46).

The Ability to Seek care was also compromised in this research as most participants did not seek health care every time they needed it. Structural barriers to health care, such as long waiting times, cancellation or postponing of medical appointments, language difficulties, and unfamiliarity with the health system, were appointed by refugees as the main reasons for not seeking care, similar to other studies on migrants and refugees’ health care (6, 12, 40, 44, 47). It is indisputable that the COVID-19 pandemic put overwhelming pressure upon health care systems, limiting their capacity to respond. This was particularly evident in Portugal, where unmet needs for medical care in the first year of the pandemic were the second highest among the Organization for Economic Co-operation and Development (OECD) countries, affecting more than a third of the population and especially impacting people in the lowest quintile of income (48). Nevertheless, the persistent nature of the health care barriers encountered by migrants and refugees is attested by their presence long before the pandemic, both globally (38), and in several Portuguese studies among migrants (12, 15, 47), suggesting that major interventions are necessary to reduce health systems inequalities.

Half of the participants were unaware of the existence of a national health line (*linha SNS24*). During the pandemic, and particularly during lockdown, this phone line was mandated as the primary contact with the national health system to alleviate pressure on health services. It acted as a source of information about preventive measures, provided case and contacts management, quarantine, isolation, and vaccination guidance, and, when applicable, access to the respective certifications of work absence. Also of particular importance, it allowed free testing to people registered in the national health system, provided the initial management of people with COVID-19 symptoms, and served as a referral system to healthcare providers according to the severity of symptoms (49). A non-COVID-19 line was also available to assess and direct people to medical consultation if justified. Lack of awareness of refugees about this telephone line may have impaired knowledge about health care options and modes of navigating the health system during the pandemic.

While the majority of healthcare providers sought by participants were family doctors, pharmacists, emergency doctors, and hospital specialist doctors, only a small percentage sought counseling services. These findings are in line with a systematic review of the underutilization and access to mental health services among refugees and asylum seekers in Europe (50). The distressing experiences faced by refugees act as risk factors for mental disease (51–53), doubling their risk of suffering from post-traumatic stress disorder and depression compared with economic migrants (50). The detrimental effect of the COVID-19 pandemic control measures on mental health further accentuated this vulnerability in asylum-seekers and other migrants (28). Moreover, increased perceptions of discrimination, socioeconomic difficulties, and unmet needs for medical care among refugees and migrants due to the pandemic also contributed to worsening their mental health outcomes (54). The discrepancy between refugees’ mental health needs and the actual mental care they receive can be attributed to several factors, rooted in the aforementioned barriers, namely language difficulties, unavailability or lack of timely appointments, unawareness regarding providers’ services, or constraints to virtual care access during the pandemic (11, 50, 52). Additionally, cultural barriers, stigma, low self-perceptions, and awareness about mental disease are also important impediments to access, probably contributing to the low rates of mental help-seeking among refugees (50, 52).

Regarding health services, most participants relied on the public sector to get health care, which is probably explained by the economic insufficiency reported in the study.

### 4.4 Availability and accommodation and ability to reach

During the pandemic, half of the respondents who contacted the healthcare center found it very difficult to get medical advice by phone or email. Restrictions on social contacts, especially during lockdowns, forced healthcare services into a fast transition to alternative and non-face-to-face modalities of contact with users. In this study, there was limited availability and accommodation of services for refugees during the pandemic, as the offered means of obtaining a medical consultation were ineffective.

When considering the ability to reach, namely the physical mobility to the health center, most participants reported that it was easy to get to the primary care center or hospital. This is probably partially because all respondents live in an urban setting, specifically in the country’s capital, where there is a greater concentration of services and resources, including public transportation. As of November 2022, of the 1,294 primary care units of the country, approximately 68% were concentrated in Porto and Lisbon regions (514 and 362 respectively) (55). Likewise, in 2021, most of the 107 country’s public hospitals were in the Lisbon Metropolitan Area (24 hospitals) (56). In a study among immigrants living in the Lisbon Metropolitan Area, the geographic proximity of the healthcare centers was found to be the main reason for their utilization (12). Another factor that may have facilitated responders’ mobility was the use of public transportation for free, as refugees are attributed a gratuitous monthly travel pass during the monitoring phase of PMAR Lx. The intent to assess the ability to reach through occupational flexibility was limited in this study, as the great majority of responders were unemployed, and out of the few who were employed, not all needed health care.

### 4.5 Affordability and ability to pay

Over half of the participants reported paying for health care, despite refugees being exempt from user fees in the SNS. The lack of affordability in this study was mainly related to paying for medication and, to a lesser extent, dental care. These findings are consistent with a study among immigrants in Portugal that showed greater financial difficulties for immigrants to acquire pharmaceuticals compared to natives (19). The Portuguese SNS covers several but limited services, namely medical appointments in primary care and specialized outpatient care, pharmaceuticals, and other services prescribed by physicians (57). Despite refugees’ entitlement to user fee exemption, which enables them to receive the aforementioned services without costs, the SNS coverage for pharmaceuticals operates under a coinsurance scheme in which a portion is paid by the user (58). In 2020, pharmaceuticals and other medical goods constituted the main reason for out-of-pocket expenditures in OECD countries, due to a lesser extent of governments’ coverage comparatively to inpatient/outpatient care. Moreover, in Portugal, coverage for pharmaceuticals was below the average of 59% of the OECD countries (59). Nevertheless, the extensive offer of generic medications should theoretically allow people with scarce economic resources to maintain their treatment at sometimes considerably lower costs. Concurrently, access to the government’s cost-sharing in the acquisition of pharmaceuticals requires the presentation of a medical prescription (which is usually provided subsequently to a medical appointment). A study of the results from the 2014 National Health Survey in Portugal showed that migrants were more likely than natives to use medications without a prescription (60). Obstacles to medical appointments observed in this study, and therefore inability to obtain a prescription, may have contributed to refugees resorting to over-the-counter pharmaceuticals (which are not covered by the SNS). Dental care is mainly provided by dentists in the private sector despite its inclusion in the SNS in 2016 (58, 61). In cases of oral cancer suspicion and some situations of social vulnerability (62), within which refugees are not included, SNS offers a dental paycheck that covers treatments free of charge (58).

Assessment of ability to pay showed that up to 40% of responders experienced times when they could not afford a medical examination/treatment, which can be understood in the context of the high percentages of unemployment and difficulty in making ends meet reported by the participants. Costs associated with dental care, medical appointments, or exams in the private sector (as an attempt to cover health needs in a timely manner), and medications were some of the cited reasons for the inability to pay.

### 4.6 Appropriateness and ability to engage

In terms of appropriateness and adequacy of services, over a third of the participants were not offered an interpreting service when receiving health care during the pandemic, even though the large majority were not proficient in Portuguese or English. In cases where interpretation was offered, it was mainly provided by the cultural mediators of the organization CRESCER, which could partially be explained by the participants’ median stay of 17 months in the country (which coincides with the monitoring phase of PMAR Lx). Lack of adequate communication between healthcare professionals and refugees leads to misunderstandings and misdiagnosis (3, 44). In addition, it generates feelings of emotional distress, distrust, and perceptions of exclusion, and propels disconnection and underutilization of services by refugees and immigrants (44, 51). Although interpretation services provided by CRESCER organization may contribute to the fulfillment of most refugees’ linguistic needs when articulating with healthcare services during the monitoring phase of the PMAR Lx, devising long-term strategies to address this issue is warranted. With the intent to bridge the communication gap between migrants and institutions, the High Commission for Migration provides a toll-free interpretation telephone service (63). Nevertheless, the line is not available 24/7 and it is not specific for health care purposes, making it unsuitable for emergencies and prone to the inadequate interpretation of medical terminology. Most participants reported that when receiving health care during the pandemic, healthcare professionals did not discuss with them treatment options or treatment side effects. Poor technical and interpersonal quality of care contributes to restricting access (2). A review of primary care access among immigrants in Canada showed that the lack of patient involvement in treatment decision-making results in service dissatisfaction and, eventually, change in healthcare providers (44). Conversely, a study with refugees and immigrants in Denmark during the pandemic underscored the importance of the coproduction of health as a means to deliver quality healthcare service to this vulnerable population, sustained by trustful relationships with healthcare providers, which enhanced patient participation in decisions as well as their overall health care (64).

The large majority of participants were vaccinated against COVID-19 and adopted preventive measures against infection, denoting significant participation in public health recommendations and an ability to engage in health care. The participants’ high level of education probably played a role in this engagement. Although there was a high percentage of vaccination at the time of the study, most participants had an incomplete schedule. The findings on following precautions against SARS-CoV2 infection in this study were similar to those in a World Health Organization worldwide survey of refugees and migrants on the self-reported impact of COVID-19 (29). In the latter, there was also a high adherence to measures such as increased hand washing, social distancing, and covering the nose and mouth. However, the ability to follow these precautions varied across regions, with refugees and migrants from Africa and Southeast Asia showing higher noncompliance percentages due to the lack of suitable living conditions (29).

## 5 Limitations

Although this research was designed to comprehensively address all dimensions of access as contemplated in Levesque’s theoretical framework, it could not encompass all the underlying determinants of access, highlighting the complexity of the subject at hand. This complexity extended to the methodology of characterizing the dimensions of the framework. The process of allocating a variable and corresponding question to a specific dimension or ability within the framework proved challenging, as some questions applied to more than one dimension or ability (65). The non-probabilistic and small sample limits the generalization of the findings, and focusing on just one refugee reception organization, to save time and resources, may have resulted in a selection bias (66). Additionally, the questionnaire refers to a timeline of more than 2 years, which may have compromised the accuracy of memories regarding the studied events and have led to a recall bias (66). Finally, the cross-sectional nature of the study does not allow for causality to be established: for that purpose, it would be necessary to carry out another type of study design (i.e., cohort study).

### 5.1 Implications for future research

Identified population characteristics and barriers to health care access in this research may inform future studies on the health care needs of refugees in Portugal and suggest how health services could be improved to meet those needs. Findings in this research also shed light on persistent challenges that require the development of strategies and policies aimed at reducing inequalities in health care access. However, a deeper understanding of the specificities of the refugee population in Portugal is essential for designing targeted interventions that facilitate access to health care. Studies with larger samples, involving more refugee hosting entities, and in different geographic locations of the country, would allow for better representativeness of the refugee population in Portugal, thus providing a more comprehensive understanding of health care access. Likewise, to better understand the complexity of health care access, it is also necessary to explore the perceptions and experiences of both refugees and healthcare providers. Qualitative studies could allow for in-depth insights into the specificities and needs of both access agents, therefore enabling effective and context-specific strategies.

## 6 Conclusion

To the extent of our knowledge, this is the first quantitative study exploring health care access among refugees in Portugal during the COVID-19 pandemic. It also provided a platform for refugees’ input on the subject, using a comprehensive framework on health care access, exploring both supply- and demand-side determinants. Although all the participants were registered in the national health system and most had received at least one dose of vaccination against COVID-19, our study also suggests constraints in several dimensions of access:- an insufficiency of inclusive communication by healthcare services/authorities, as language-appropriate information about COVID-19 did not reach all of the participants;- a paucity of cultural competence, as more than half of the refugees felt like healthcare professionals did not always show respect towards their culture; −an underutilization of mental health services, considering the minority of refugees that sought counseling; −a lack in the coproduction of health care, as the majority of refugees felt they were not involved in their health care process decision making. Table 4 summarizes possible policy implications from the aforementioned barriers. The outcomes of this study will potentially make visible difficulties refugees experience in health care access and thus inform future studies with larger and representative samples. Vertical health policies with multilevel strategies are essential to improve access to health care by refugees. To this end, the establishment of work alliances with refugee communities’ representatives would be an unvaluable asset to ensure an open channel of communication with the target population and the alignment of measures with people’s specific needs.

**Table 4:** Extracted policy implications from the study.

Potential Health Care Access Areas to Improve		Possible Interventions	
*Inclusive communication*	*Close Collaboration with Refugees’representatives*	Work with translators/interpreters in: - developing culturally adapted communication materials on health care functioning and individual rights to health in the host country - choosing the most widely used communication channels by the communities to disseminate information - developing Standard Operating Procedures for risk communication in public health emergencies with a defined chain of command within the communities Generalize the inclusion of interpreters in health care settings	*Coproduction of Health*
*Cultural competence of healthcare providers*		Invest in training courses for healthcare professionals on cultural diversity awareness and cultural competence skills development Integration of cultural diversity awareness in academic curricula of health care professionals Promote cultural exchange opportunities between refugees and healthcare professionals through events or training courses Include Cultural Mediators in health care settings as permanent staff	
*Utilization of counseling services*		Use appropriate channels to disseminate information about counseling services Promote information sessions on mental health: increase refugees’ self-perceptions about mental health and reduce stigma associated with mental disease	

## Data availability statement

The original contributions presented in the study are included in the article/Supplementary material, further inquiries can be directed to the corresponding author.

## Ethics statement

The studies involving humans were approved by Ethics Committee of Institute of Hygiene and Tropical Medicine of New University of Lisbon. The studies were conducted in accordance with the local legislation and institutional requirements. The participants provided their written informed consent to participate in this study.

## Author contributions

VP: Conceptualization, Data curation, Formal analysis, Investigation, Methodology, Resources, Supervision, Visualization, Writing – original draft, Writing – review & editing. SH: Conceptualization, Methodology, Supervision, Writing – review & editing. MO: Conceptualization, Data curation, Funding acquisition, Methodology, Project administration, Resources, Supervision, Writing – review & editing.
