# Supplementary material for: Exploring refugees’ health care access in times of COVID-19: a quantitative study in the Lisbon region, Portugal
Source: Front Public Health. 2024 Jan 29;12:1337299. doi: 10.3389/fpubh.2024.1337299 (PMC10859453; doi:10.3389/fpubh.2024.1337299)
Supplement: Supplementary file 1 [file Table_1.DOCX]

| **APPENDIX I. 1^st^ domain: sociodemographic-, migration-, healthcare services-, and COVID-19-related variables** | | | |
| --- | --- | --- | --- |
| **VARIABLE** | **VARIABLE TYPE** | **ANSWER CATEGORIES/DESCRIPTORS** | **REF.** |
| SOCIODEMOGRAPHIC DETAILS | | | |
| **GENDER** | Categorical, nominal | female, male, other, decline to answer | - |
| **AGE** | Quantitative, discrete | years, decline to answer | - |
| **HIGHEST EDUCATIONAL ATTAINMENT** | Categorical, ordinal | no formal education, primary school, secondary school, post-secondary/tertiary school or above, decline to answer | (32) |
| **MARITAL STATUS** | Categorical, nominal | single, married/consensual union, divorced/separated, widowed, decline to answer | (12) |
| **RELIGION** | Categorical, nominal | Islam, Christianity, Judaism, no religion, other, decline to answer | (12)  (15) |
| **EMPLOYMENT STATUS** | Categorical, nominal | employed, student, retired, housekeeper, unemployed, other, decline to answer | (12) |
| **NUMBER OF PEOPLE IN HOUSEHOLD** | Quantitative, discrete | number of people, decline to answer | (32)  (33) |
| **PERCEIVED HOUSEHOLD INCOME** | Qualitative, ordinal | easily, fairly easily, with some difficulty, with great difficulty, don’t know, decline to answer | (65) |
| MIGRATION DETAILS | | | |
| **COUNTRY OF ORIGIN** | Categorical, nominal | country of origin, decline to answer | (45)  (27) |
| **INTEGRATION IN THE REFUGEE RECEPTION PROGRAM** | Categorical, nominal | yes, no, don’t know, decline to answer | - |
| **LENGTH OF STAY IN PORTUGAL (mo)** | Quantitative, discrete | number of months, don’t know, decline to answer | (12)  (15) |
|  |  |  |  |

| **APPENDIX I. 1^st^ domain: sociodemographic-, migration-, healthcare services-, and COVID-19-related variables *(cont.)*** | | | |
| --- | --- | --- | --- |
| **VARIABLE** | **VARIABLE TYPE** | **ANSWER CATEGORIES/DESCRIPTORS** | **REF.** |
| **NATIVE LANGUAGE** | Qualitative, nominal | language spoken, decline to answer | (66) |
| **PERCEIVED PROFICIENCY IN PORTUGUESE** | Qualitative, ordinal | very well, well, not well, not at all, decline to answer | (67) |
| HEALTHCARE SERVICES | | | |
| **REGISTRATION IN A PRIMARY CARE CENTER** | Categorical, nominal | yes, no, decline to answer | (15) |
| **HEALTHCARE UTILIZATION** | Categorical, nominal | yes, no, decline to answer | - |
| COVID-19 INFORMATION | | | |
| **POSITIVE TEST TO SARS-COV2** | Categorical, nominal | yes, no, never been tested, decline to answer | (68) (27) |
| **HOSPITALIZATION DUE TO COVID-19** | Categorical, nominal | yes, no, not applicable (never been tested / never tested positive), decline to answer | (69)  (68) |
| **VACCINATION AGAINST COVID-19** | Categorical, nominal | yes, all doses required, yes, but not all doses required, no, decline to answer | - |
| **PREVENTIVE MEASURES SARS-CoV-2** | Categorical, nominal | washing your hands for 20 secs with soap and water, use sanitizers, wear face mask when in closed public spaces, keep social distance, taking over the counter medicines, change diet, other, not applicable (did not practice any protective measures), decline to answer | (70)  (27) |
|  |  |  |  |

| **APPENDIX 2. 2^nd^ domain: dimensions of accessibility of services and abilities of persons** | | | | | |
| --- | --- | --- | --- | --- | --- |
| **VARIABLE** | **ACCESS COMPONENT** | **DIMENSION** | **VARIABLE TYPE** | **ANSWER CATEGORIES/DESCRIPTORS** | **REF.** |
| **ACCESS TO INFORMATION ABOUT COVID-19 IN UNDERSTANDABLE LANGUAGE** | **ACCESSIBILITY OF SERVICES** | Approachability | Categorical, nominal | yes, no, decline to answer | - |
| **SOURCE OF INFORMATION ON COVID-19** | **ABILITIES OF PERSONS** | To perceive | Categorical, nominal | news from country of origin, news from country where I live (PT), social media, friends or family, place of worship, healthcare professionals, non-governmental organizations, other, decline to answer | (12) (33) (27)  (70) |
| **KNOWLEDGE OF SYMPTOMS OF COVID-19** | **ABILITIES OF PERSONS** | To perceive | Categorical, nominal | fever or chills, difficulty breathing or cough, fatigue, muscle or body aches, new loss of taste or smell, sore throat, congestion or runny nose, nausea or vomiting, diarrhea, constipation, bleeding, decline to answer (true, false, don’t know) | (33) (24)  (70) |
| **ASYMPTOMATIC SPREAD OF COVID-19** | **ABILITIES OF PERSONS** | To perceive | Categorical, nominal | true, false, don´t know, decline to answer | (33) |
| **PREVENTION OF COVID-19 BY EATING SPICY FOOD** | **ABILITIES OF PERSONS** | To perceive | Categorical, nominal | true, false, don´t know, decline to answer | (33) (32) |
| **NEEDED HEALTH CARE DURING THE PANDEMIC** | **ABILITIES OF PERSONS** | To perceive | Categorical, nominal | yes, no, decline to answer | (66) |
| **CULTURAL COMPETENCE IN HEALTH CARE PROVIDED** | **ACCESSIBILITY OF SERVICES** | Acceptability | Categorical, nominal | always, sometimes, rarely, never, not applicable (did not receive health care), decline to answer | (63) |
| **SOUGHT HEALTH CARE DURING THE PANDEMIC** | **ABILITIES OF PERSONS** | To seek | Categorical, nominal | yes, no, not applicable (did not need health care), decline to answer | (66)  (68) |

| **APPENDIX 2. 2^nd^ domain: dimensions of accessibility of services and abilities of persons *(cont.)*** | | | | | |
| --- | --- | --- | --- | --- | --- |
| **VARIABLE** | **ACCESS COMPONENT** | **DIMENSION** | **VARIABLE TYPE** | **ANSWER CATEGORIES/DESCRIPTORS** | **REF.** |
| **MEDICAL APPOINTMENT/PERFORM EXAMS DURING WORKING HOURS** | **ABILITIES OF PERSONS** | To reach | Categorical, nominal | yes, no, don’t know (never needed health care during working hours), not applicable (don’t work), decline to answer | (72) |
| **PAY FOR HEALTHCARE SERVICES** | **ACCESSIBILITY OF SERVICES** | Affordability | Categorical, nominal | yes, no, not applicable (did not need/receive health care), decline to answer | (63) |
| **COULD NOT AFFORD MEDICAL EXAMINATION/TREATMENT** | **ABILITIES OF PERSONS** | To pay | Categorical, nominal | yes, no, not applicable (did not need medical examination /treatment), decline to answer | (69)  (63) |
| **INTERPRETING SERVICE** | **ACCESSIBILITY OF SERVICES** | Appropriateness | Categorical, nominal | yes, no, not applicable (never received health care/ did not need interpretation), decline to answer | - |
| **DISCUSSION OF TREATMENT OPTIONS/SIDE EFFECTS** | **ACCESSIBILITY OF SERVICES** | Appropriateness | Categorical, nominal | always, sometimes, rarely, never, not applicable (did not receive health care), decline to answer | (63) |
| **VACCINATION AGAINST COVID-19** | **ABILITIES OF PERSONS** | To engage | Categorical, nominal | yes, all doses required, yes, but not all doses required, no, decline to answer | - |
| **PREVENTIVE MEASURES SARS-COV2** | **ABILITIES OF PERSONS** | To engage | Categorical, nominal | washing your hands for 20 secs with soap and water, use sanitizers, wear face mask when in closed public spaces, keep social distance, taking over the counter medicines, change diet, other, not applicable (did not practice any protective measures), decline to answer | (70) |
